# Pregnancy and Lactation in Sprague-Dawley Rats Result in Permanent Reductions of Tibia Trabecular Bone Mineral Density and Structure but Consumption of Red Rooibos Herbal Tea Supports the Partial Recovery

**DOI:** 10.3389/fnut.2021.798936

**Published:** 2021-12-07

**Authors:** Michael D. McAlpine, Jenalyn L. Yumol, Wendy E. Ward

**Affiliations:** ^1^Department of Kinesiology, Faculty of Applied Health Sciences, Brock University, St. Catharines, ON, Canada; ^2^Centre for Bone and Muscle Health, Brock University, St. Catharines, ON, Canada

**Keywords:** bone, red rooibos, polyphenols, pregnancy, lactation

## Abstract

During pregnancy and lactation, maternal bone mineral density (BMD) is reduced as calcium is mobilized to support offspring bone development. In humans, BMD returns to pre-pregnancy levels shortly after delivery, shifting from a high rate of bone resorption during pregnancy and lactation, into a rapid phase of bone formation post-lactation. This rapid change in bone turnover may provide an opportunity to stimulate a greater gain in BMD and stronger trabecular and cortical structure than present pre-pregnancy. Providing polyphenols present in red rooibos herbal tea may promote such an effect. *In vitro*, red rooibos polyphenols stimulate osteoblast activity, reduce osteoclastic resorption, and increase mineral production. The study objective was to determine if consuming red rooibos from pre-pregnancy through to 4 months post-lactation resulted in a higher BMD and improved trabecular and cortical bone structure in a commonly used rat model. Female Sprague-Dawley rats (*n* = 42) were randomized to one of the following groups: PREG TEA (pregnant, received supplemental level of red rooibos in water: ~2.6 g /kg body weight/day in water), PREG WATER (pregnant, received water), or NONPREG CON (age-matched, non-pregnant control, received water) from 2 weeks pre-pregnancy (age 8 weeks) through to 4 months post-lactation. Rats were fed AIN-93G (pre-pregnancy through to the end of lactation) and AIN-93M (post-lactation onwards). BMD and trabecular structure (bone volume fraction, trabecular number, trabecular separation) were improved (*p* < 0.05) by 1- or 2-months post-lactation when comparing PREG TEA to PREG CON, though neither group recovered to the level of NONPREG CON. Cortical outcomes (cortical area fraction, cortical thickness, tissue mineral density) for PREG TEA and PREG CON were reduced (*p* < 0.05) following lactation but returned to the level of NONPREG CON by 2-months post-lactation, with the exception of cortical thickness. The lack of recovery of BMD and key outcomes of trabecular bone structure was unexpected. While consumption of red rooibos did not result in stronger bone post-lactation, red rooibos did support the partial recovery of trabecular BMD and bone structure following pregnancy and lactation. The findings also provide insight into the timing and dose of polyphenols to study in future interventions.

## Introduction

In humans, there is a decrease in BMD and trabecular and cortical structure during pregnancy and lactation due to an increased demand in mobilized calcium for offspring bone development (1). Reductions in BMD during human pregnancy and lactation, measured using dual energy X-ray absorptiometry (DXA), have been reported to be 5–10% (1, 2) and it is believed that women typically return to their pre-pregnancy BMD after delivery as number of pregnancies and duration of breast-feeding is not considered a risk for developing osteoporosis later in life (3–5). More specifically, to meet the elevated calcium demand by the fetus, intestinal calcium absorption is increased beginning in the first trimester. In humans, intestinal calcium absorption returns to pre-pregnancy levels during lactation (but stays elevated in rodents) (2) while there is a concurrent increase in skeletal resorption to provide calcium for offspring bone growth for both humans and rodents (6). This results in an uncoupling of bone turnover with elevated levels of bone resorption compared to formation leading to reductions in BMD and trabecular and cortical structure observed in both humans and rodents. Despite the reduction in BMD following lactation, it is transient as an uncoupling of bone turnover persists but it is reversed with greater formation than resorption occurring (7–9). These high rates of bone turnover observed during pregnancy, lactation, and recovery may provide a “window of opportunity” to stimulate a greater gain in BMD and stronger trabecular and cortical structure than was present pre-pregnancy.

A key contributing factor to the promotion of bone health is diet. Several nutrients, including calcium and vitamin D, as well as various foods and food components have been studied for their bone promoting or supporting effects (10–13). Tea and its polyphenols–including some herbal teas such as red rooibos (RR)-may promote bone health and mineral production (14–17). Several epidemiological studies in different countries including Australia, Britain, and Taiwan have also identified positive associations between black or green tea consumption and greater BMD later in life (18–20). To date, RR tea has not been studied *in vivo* for potential bone promoting or supporting effects. Also, *in vitro*, a wide variety of teas derived from *Camellia sinensis* and herbal teas from other plants such as RR have been shown to increase osteoblast activity and proliferation (16, 17); while also having the capacity to decrease osteoclast activity and proliferation (21, 22)–possibly by acting as antioxidants and thereby reducing reactive oxygen species (ROS). ROS have been shown to suppress mineralization and increase resorption *in vitro* (23–25). Moreover, metabolism is elevated during pregnancy and lactation, along with ROS, due to the requirements for developing fetal tissues (26). Reduction of ROS by tea and its respective polyphenols may counter these effects leading to greater mineralization by osteoblasts and a reduction of osteoclast resorption.

Commonly consumed teas, such as green and black teas, are known to contain caffeine and thus would not be recommended for pregnant women while red rooibos (RR) tea does not contain caffeine. RR herbal tea originates from the *Aspalathus linearis* plant. It is fully oxidized and has a unique profile of polyphenols-including aspalathin, aspalalinin, and nothofagin-that are not present in other teas. Previously our lab has shown RR to have the capacity *in vitro* to increase mineralization by osteoblasts (Saos-2 cells) in a dose-dependent manner (16) using levels that can be achieved by consuming several cups of RR tea a day through to supplementation. Improved cell activity was also observed. With respect to bone resorption, RR has been shown to reduce osteoclast formation and activity *in vitro* using RAW264.7 cells, and displayed oxidant scavenging activity without any cytotoxic effects (27). Although it is likely that these polyphenols will be altered upon absorption and digestion it is possible that their metabolized forms may also have positive effects on bone. Taken together, an increase in mineralization and concurrent reduction in resorption would lead to an increase in bone formation which may support the acquisition of greater BMD and structure following pregnancy and lactation.

The objective of this study was to determine if continuous consumption of RR tea from pre-pregnancy through to 4 months post-lactation resulted in higher BMD and improved structure of trabecular and cortical bone in the tibia compared to a water control. It was hypothesized that consumption of RR tea during pregnancy, lactation, and recovery would result in greater BMD and improved structure of the tibia compared to consumption of only water.

## Materials and Methods

### Animals and Diets

Forty-two female and fourteen male Sprague-Dawley rats (5 weeks of age) were purchased from Charles Rivers Laboratories (St. Constant, QC, Canada). Rats were singly housed under a controlled environment (20°C, and 12 h light and dark cycles) and supplied with water and diet (AIN-93G, Envigo; Indianapolis, IN, USA) *ad libitum*. Each rat had access to physical enrichment in their cage including crinkle nest and a red rat retreat to provide shelter and lower stress. Body weight was measured weekly while diet and water intake was measured bi-weekly using an electronic scale.

### Experimental Design

Several methodological aspects were considered in planning and conducting the study to help ensure reproducibility (Supplementary Material 1). Following 1 week of acclimatization to handling and the environment of the animal facility, female rats were randomly assigned to one of three groups (*n* = 14/group): a pregnancy and lactation group receiving RR tea prepared in water (PREG TEA), a pregnancy and lactation group receiving only water (PREG CON) or an age matched control group that was not mated (NONPREG CON). An *a priori* sample size analysis was conducted using findings from previous literature that assessed the effects of green tea polyphenols on bone in response to LPS induced chronic bone inflammation and bone loss with a primary outcome of tibial BV/TV used (28)-a sample size of 6 was calculated to be necessary for the study (Supplementary Material 2). An additional 8 rats per group were included if pregnancy was not achieved in all rats, a healthy litter with a minimum of 10 pups was not delivered, or if there were complications with longitudinal scans (i.e., death due to anesthetic). As well, pregnancy does not exert as much systematic stress on the skeletal system as LPS induced inflammation so it was anticipated that differences between intervention groups and control could be attenuated compared to this previously published trial. Rats in the PREG CON and NONPREG CON groups had *ad libitum* access to AIN-93G diet and water, while those in the PREG TEA group received AIN-93G diet and RR tea *ad libitum* (concentration of approximately 2.6 g of RR/kg of body weight per day). This concentration is comparable to consuming approximately 12 cups of RR tea daily and was calculated through the conversion of a human equivalent dose (HED) to an animal equivalent dose (AED) (29) (Supplementary Material 3). This concentration was chosen based on previous research demonstrating greater concentrations of RR tea eliciting greater levels of mineralization *in vitro* (16). For the following 2 weeks, rats were further acclimatized to the facility and their respective group while water intake was measured to ensure rats in PREG TEA did not have an aversion to the taste. After a 3 week period of acclimatization to diet and environment, half of the rats from each of the PREG TEA and PREG CON groups were mated one to one with males of the same age. The remaining rats from each of these groups were mated 2 weeks later with the same group of males to ensure any potential paternal differences were equally distributed between groups (Supplementary Material 4). The staggered mating also helped with time management, allowing for the longitudinal *in vivo* scans throughout the study. The time involved with *in vivo* scanning would have made it challenging to study rats as one cohort. Of the initial 14 rats randomized to the PREG CON group, one rat did not become pregnant after 2 estrous cycles and was removed from the study and one rat did not recover from anesthesia following their post-lactation scan (missing values were replaced by mean imputation). This resulted in the following sample sizes (*n*): PREG TEA = 14, PREG CON = 13, and NONPREG CON = 14. At postnatal day 3 (PND 3), litters were culled to 5 male and 5 females to normalize milk production among dams. Pups were selected to be culled based on their weight in comparison with the average weight of their litter–with pups that had the greatest deviation from the group mean being culled. Litter weight and average pup weight was measured at PND 3 (prior to culling), and post-culling at PND 7, 14, and 21. Pups were weaned at PND 21. From the end of lactation through 4 months post-lactation, dams were fed AIN-93M (Envigo; Indianapolis, IN, USA). Due to the lack of a litter in rats from NONPREG CON and the need to protect the RR tea from light by wrapping drinking bottles in aluminum foil, experimenters were not blinded to groups during the *in vivo* portion of the trial. The protocol (#18-03-02) was approved by the Animal Care Committee at Brock University.

### Preparation of Red Rooibos Herbal Tea and Measurement of Total Polyphenol Content

Loose leaf RR tea was prepared twice weekly (every 3 or 4 days) following manufacturer's recommended steeping time and temperature. RR tea was weighed to the appropriate amount and transferred to tea bags (~5g of tea/bag) to mimic what would normally be consumed in humans. Tea bags were then placed in glass beakers and steeped for 5 min in water that was 96°C at the onset of steeping. Following 5 min of steeping, tea bags were removed and the resulting RR was cooled to room temperature. Once cooled, all beakers of RR were combined to ensure a homogenous mixture. To ensure rats received approximately 2.6 g of RR/kg of body weight, the concentration of RR tea was constantly adjusted depending on both average body weight and water intake from the previous week's measurements. This allowed a consistent intake of RR tea relative to body weight throughout the study despite any changes in water intake while also allowing rats *ad libitum* access. An example scenario and calculation is shown in Supplementary Material 5. Total polyphenol content (TPC) of RR tea was measured throughout the study using Folin-Ciocalteau's reagent and gallic acid as a standard according to ISO 14502-1 as previously reported (30).

### *In vivo* μCT Scanning of Tibia

**Figure 1:**
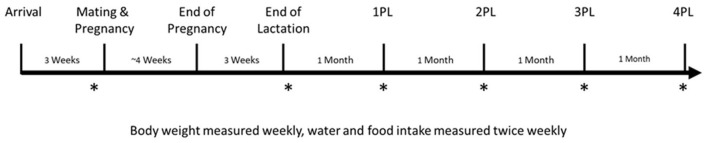
Study design. Female Sprague-Dawley rats arrived at 5 weeks of age and were randomized to either a PREG TEA, PREG CON, or NONPREG CON group (*n* = 14) following 1 week of acclimatization. Rats were then further acclimatized for another 2 weeks to their respective groups. Following acclimatization, females were mated for a duration of 2 estrous cycles (5 days per cycle). *In vivo* scans for measurement of bone structure and quantity of the right proximal tibia using μCT are denoted by an asterisk (*) and were completed at pre-pregnancy (Pre), immediately after lactation (PL), and 1, 2, 3, and 4 months post-lactation (1PL, 2PL, 3PL, 4 PL). Rats from NONPREG CON were scanned at the same time points to provide an age-matched control. Necropsy and organ collection occurred approximately 1 week following the 4PL scan.

The right tibia of rats were scanned at 6 time points using high resolution *in vivo* micro computed tomography (μCT) (SkyScan 1176, Bruker microCT, Belgium): prior to mating (5–7 days before the initiation of mating), immediately following the end of lactation (within 48 h), and at 1, 2, 3, and 4 months post-lactation (denoted as Pre, PL, 1PL, 2PL, 3PL, and 4PL, respectively, or at an identical age for NONPREG CON group). Prior to scanning, rats were anesthetized with isoflurane. Rats were placed in an induction chamber with a steady flow rate of approximately 2% isoflurane and anesthesia was confirmed by the absence of a response to a toe pinch. Rats were then transferred and placed in supine position on the scanning bed and isoflurane was given by nose cone to ensure adequate anesthesia to help prevent movement during the scan (31). All scans were performed with parameters that have previously been shown by our lab to be safe for longitudinal measurements–both in terms of the recovery of the rat from anesthetic between scans and without causing radiation damage to bone structure (18 μm voxel size, 1 mm aluminum filter, 700 ms exposure time, 60 kV of voltage, 200 uA of amperage, a rotation step of 0.5° over a 360° scanning frame) (31, 32). Monthly, *in vivo* scans of rat tibias using the same machine have previously shown no detrimental effects of repeated irradiation to BMD or bone structure (32). At each time-point, scanning order was determined by alternating intervention groups until all scans were complete to minimize the potential for any variability in the X-ray source when scanning. Following the 4PL scan (or age matched equivalent) rats were anesthetized with isoflurane in an induction chamber and euthanized by CO_2_ asphyxiation. Tibias were collected, weighed by digital scale, and stored. Organ weights were measured as a preliminary sign of the possibility of any toxicological effects. Study design is shown in Figure 1.

### Image Reconstruction and Analysis

Following acquisition of all scans, images were reconstructed using a Gaussian filter under the same parameters to ensure accurate comparisons. The region of interest (ROI) for analysis of trabecular bone began 150 slices (2.64 mm) distal from the point where the growth plate and the metaphysis of the tibia met and spanned 75 slices (3.96 mm) distally. The ROI for cortical bone began 400 slices (7.04 mm) distal from the point where the growth plate and the metaphysis of the tibia met and spanned 100 slices (8.80 mm) distally. Within each ROI, the distinction between trabecular and cortical bone was performed manually by the same individual (MDM) and saved as a distinct ROI for analysis. Images were first binarized using global thresholding (trabecular bone: 42–255, cortical bone: 63–255). Following binarization, images underwent several morphological operations to ensure that only bone tissue was being analyzed. Trabecular bone was analyzed for the following structure outcomes: bone volume fraction (BV/TV), trabecular thickness (Tb.Th), trabecular spacing (Tb.Sp), trabecular number (Tb.N), and bone mineral density (BMD). Cortical bone was analyzed for the following structure outcomes: cortical area fraction (Ct.Ar/Tt.Ar) periosteal perimeter (Ps.Pm), cortical thickness (Ct.Th), endocortical perimeter (Ec.Pm), marrow area (Ma.Ar), and tissue mineral density (TMD). Specific task lists for analysis of trabecular and cortical bone are shown in Supplementary Materials 6, 7, respectively.

### Statistical Analysis

The effect of group (three levels: PREG TEA, PREG CON, and NONPREG CON), time, and the interaction on food and water intake, body weight, and bone outcomes were evaluated through a mixed ANOVA with repeated measures using SPSS Statistics (v. 26, IBM). Differences between means were deemed significantly different if p < 0.05. When a significant interaction was identified a Bonferroni *post-hoc* was performed to test the main effects between PREG TEA, PREG CON, and NONPREG CON at each time point. In the case of missing values (μCT data for rat which did not recover from anesthesia following post-lactation scan), series mean imputation was performed. Potential differences in litter characteristics were assessed by *T*-tests using GraphPad Prism™ V5 (La Jolla, CA, USA).

## Results

### Food and Water Intake, Body and Organ Weights, and Litter Characteristics

**Figure 2:**
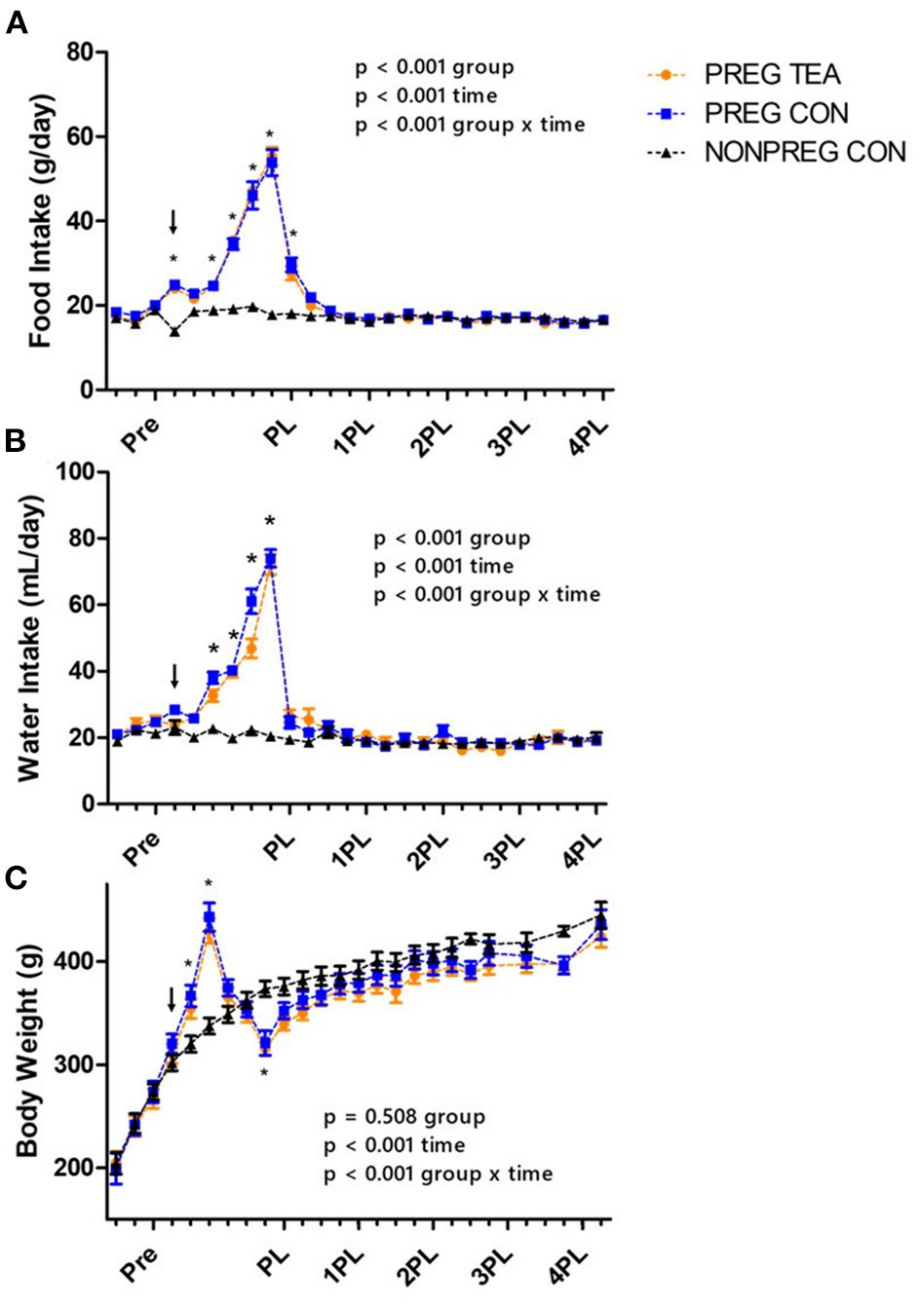
Group daily food and water intake and average body weights; **(A)** mean daily food intake, **(B)** mean daily water intake, and **(C)** body weight of female Sprague-Dawley Values are mean ± SEM, *n* = 13 (PREG CON) or 14/group (PREG TEA and NONPREG CON). Significant differences (*p* < 0.05) between pregnancy and lactation groups (PREG TEA and PREG CON) and NONPREG CON within a time point are denoted by *. The arrow above data points represents the onset of pregnancy. Pre, pre-pregnancy; PL, post-lactation; 1PL, 1 month post-lactation; 2PL, 2 months post-lactation; 3PL, 3 months post-lactation; and 4PL, 4 months post-lactation.

A significant interaction (*p* < 0.001) was observed for food intake with a significant increase during pregnancy and lactation for PREG TEA and PREG CON (Figure 2A). There was a significant interaction (*p* < 0.001) and main effects for time (*p* < 0.001) and group (*p* < 0.001) for water intake (Figure 2B). At weeks 4 and 6 through 10 of the study there was a significant increase in water intake for PREG TEA and PREG CON when compared to NONPREG CON. The average intake of RR was calculated to be 2.66 g/kg of body weight per day for rats in the PREG TEA group and the measured TPC of RR tea was determined to be 12.20 ± 0.69 mg gallic acid equivalents/g of tea (*n* = 25). For body weight there was a significant interaction (*p* < 0.001) and main effect of time (*p* < 0.001). Significant increases in body weight occurred at weeks 5 and 6 (during lactation) and a significant reduction was observed at week 9 (following delivery) (Figure 2C). There were no significant differences in kidney weight (left or right) or liver weight between the two groups at endpoint when normalized to body weight (Table 1). For litter characteristics, there were no differences in litter sizes, the proportion of males and females within each litter, and average pup weights at 3, 7, 14, and 21 days of age (Table 1).

**Table 1:** Litter characteristics and maternal organ weights at 4 months post-lactation.

	**PREG TEA**	**PREG CON**	***p*-value**
**Litter Size (*n*)^‡^**	13, 2	15, 2	0.114
# of males *(% of total*)	7, 2(*54*)	7, 2*(47*)	0.854
# of females (*% of total*)	6, 2(*46*)	8, 2*(53*)	0.086
Average Pup Weight−3 Days Old (g)	13.09, 0.49	12.71, 0.35	0.539
Average Pup Weight−7 Days Old (g)	23.74, 0.75	22.64, 0.66	0.284
Average Pup Weight−14 Days Old (g)	41.41, 1.24	41.83, 1.34	0.819
Average Pup Weight−21 Days Old (g)	65.37, 1.62	66.95, 1.97	0.540
L. Kidney Weight (mg/g of body weight)	2.90, 0.22	2.84, 0.27	0.477
R. Kidney Weight (mg/g of body weight)	2.95, 0.27	2.89, 0.26	0.559
Liver Weight (mg/g of body weight)	32.21, 1.92	34.18, 3.34	0.072

‡ *Litter size at delivery*.

### *In vivo* Measurements of Tibia Trabecular BMD and Structure

**Figure 3:**
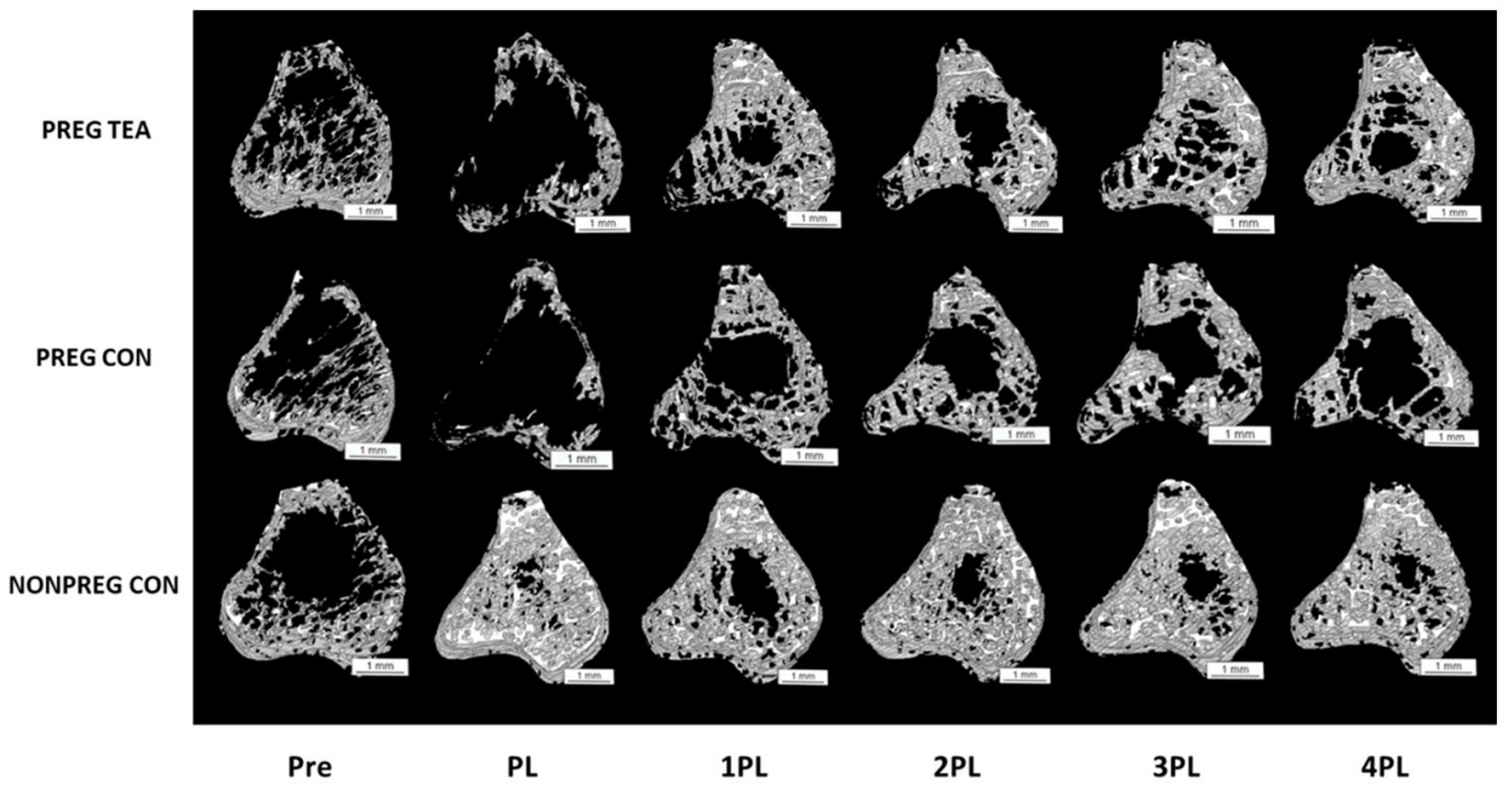
Representative 3D images of trabecular bone from right proximal tibia in female Sprague-Dawley rats pre-pregnancy, post-lactation, and 1, 2, 3, and 4 months post-lactation. Representative scans for each group are of the same rat at each time point and images were chosen by selecting the rat with the closest value for BV/TV to the group mean. Pre, pre-pregnancy; PL, post-lactation; 1PL, 1 month post-lactation; 2PL, 2 months post-lactation; 3PL, 3 months post-lactation; and 4PL, 4 months post-lactation. The white bar under each scan represents a length of 1 mm.

**Figure 4:**
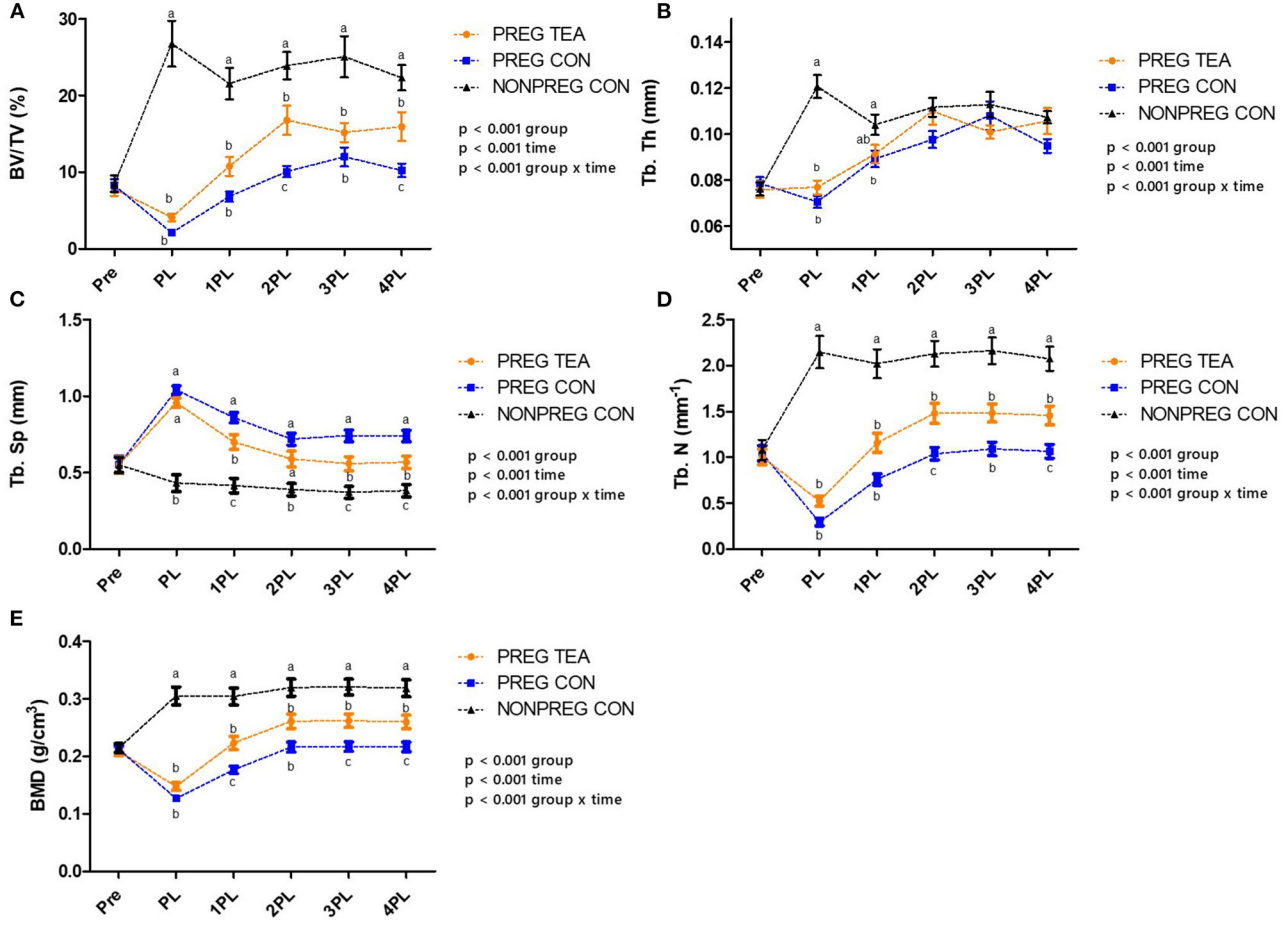
Comparisons of trabecular outcomes for PREG TEA, PREG CON, and NONPREG CON; **(A)** BV/TV, **(B)** Tb. Th, **(C)** Tb. Sp, **(D)** Tb. N, and **(E)** BMD. Values are mean ± SEM, *n* = 13 or 14/group. Differing letters denote a significant difference among groups within a time point. Pre, pre-pregnancy; PL, post-lactation; 1PL, 1 month post-lactation; 2PL, 2 months post-lactation; 3PL, 3 months post-lactation; and 4PL, 4 months post-lactation.

There was a significant interaction (*p* < 0.05) for all trabecular outcomes measured (Figures 3, 4). As a result of pregnancy and lactation, BV/TV was significantly reduced (*p* < 0.05) in both PREG TEA and PREG CON groups for the remainder of the study compared to NONPREG CON; while at 2PL and 4PL rats in PREG TEA had significantly greater (p <0.05) BV/TV than PREG CON (Figure 4A). Tb.Th was significantly (*p* < 0.05) reduced in PREG TEA and PREG CON following pregnancy and lactation but had recovered to the levels of NONPREG CON by 2PL, with PREG TEA recovering more rapidly (Figure 4B). Trabecular separation (Tb.Sp.) was significantly (*p* < 0.05) increased post-lactation in PREG TEA and PREG CON which persisted till the end of the study. Tb.Sp was significantly lower in PREG TEA than PREG CON at 1, 3, and 4PL (Figure 4C). Trabecular number (Tb.N.) was reduced (*p* < 0.05) in PREG TEA and PREG CON following lactation compared to NONPREG CON and only partially recovered by the end of the study; while PREG TEA was significantly (*p* < 0.05) greater than PREG CON at 2 and 4PL (Figure 4D). BMD was significantly (*p* < 0.05) reduced in PREG TEA and PREG CON beginning at PL and persisted for the remainder of the study, while BMD for PREG TEA was significantly (*p* < 0.05) greater than PREG CON at 1, 3, and 4PL (Figure 4E).

### *In vivo* Measurements of Tibia Cortical Tissue Mineral Density (TMD) and Structure

**Figure 5:**
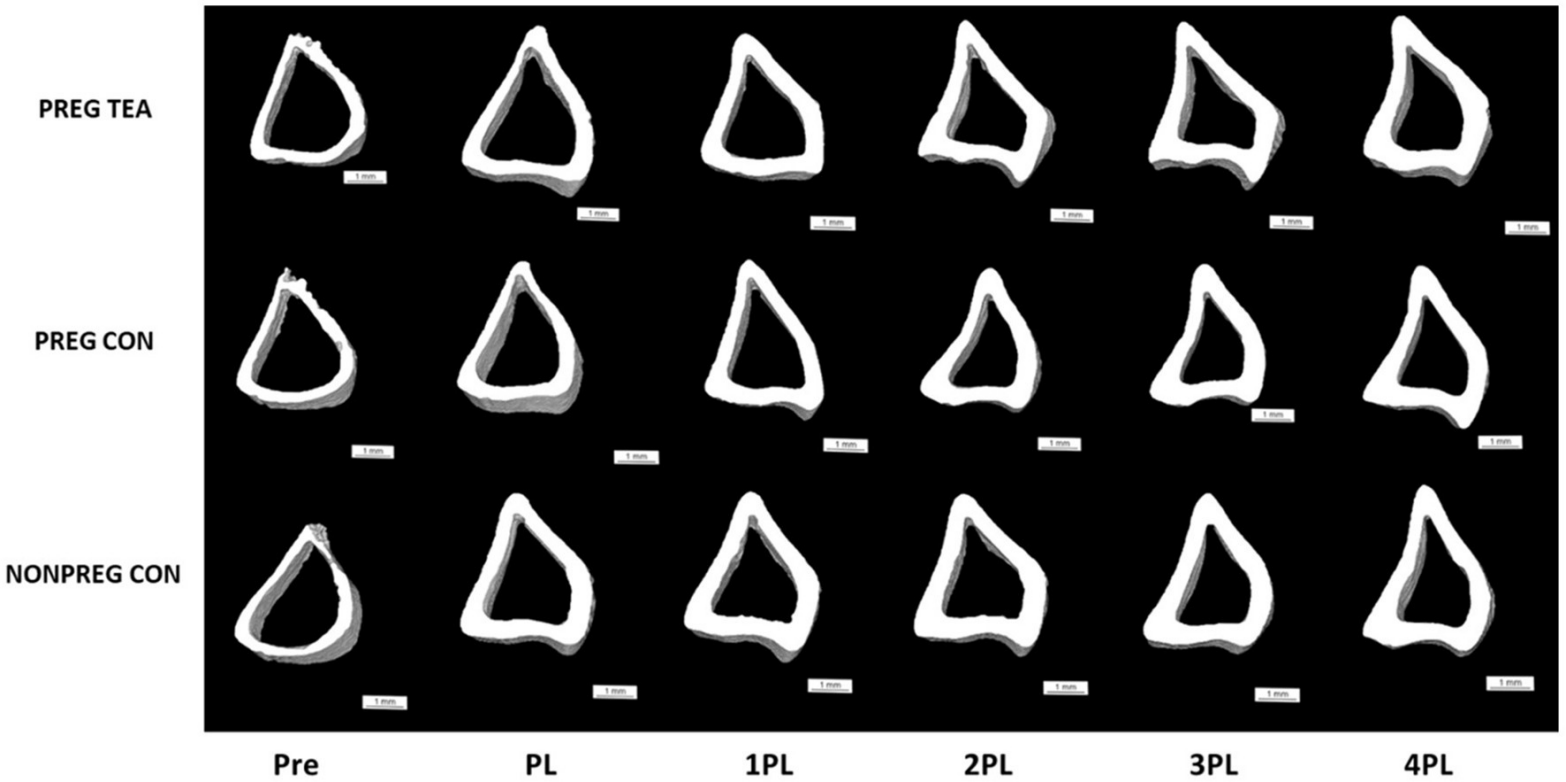
Representative 3D images of cortical bone from right proximal tibia in female Sprague-Dawley rats pre-pregnancy, post-lactation, and 1, 2, 3, and 4 months post-lactation. Representative scans for each group are of the same rat at each time point and images were chosen by selecting the rat with the closest value for Ct.Ar/Tt.Ar to the group mean. Pre: pre-pregnancy, PL, post-lactation; 1PL, 1 month post-lactation; 2PL, 2 months post-lactation; 3PL, 3 months post-lactation; and 4PL, 4 months post-lactation. The white bar under each scan represents a length of 1 mm.

**Figure 6:**
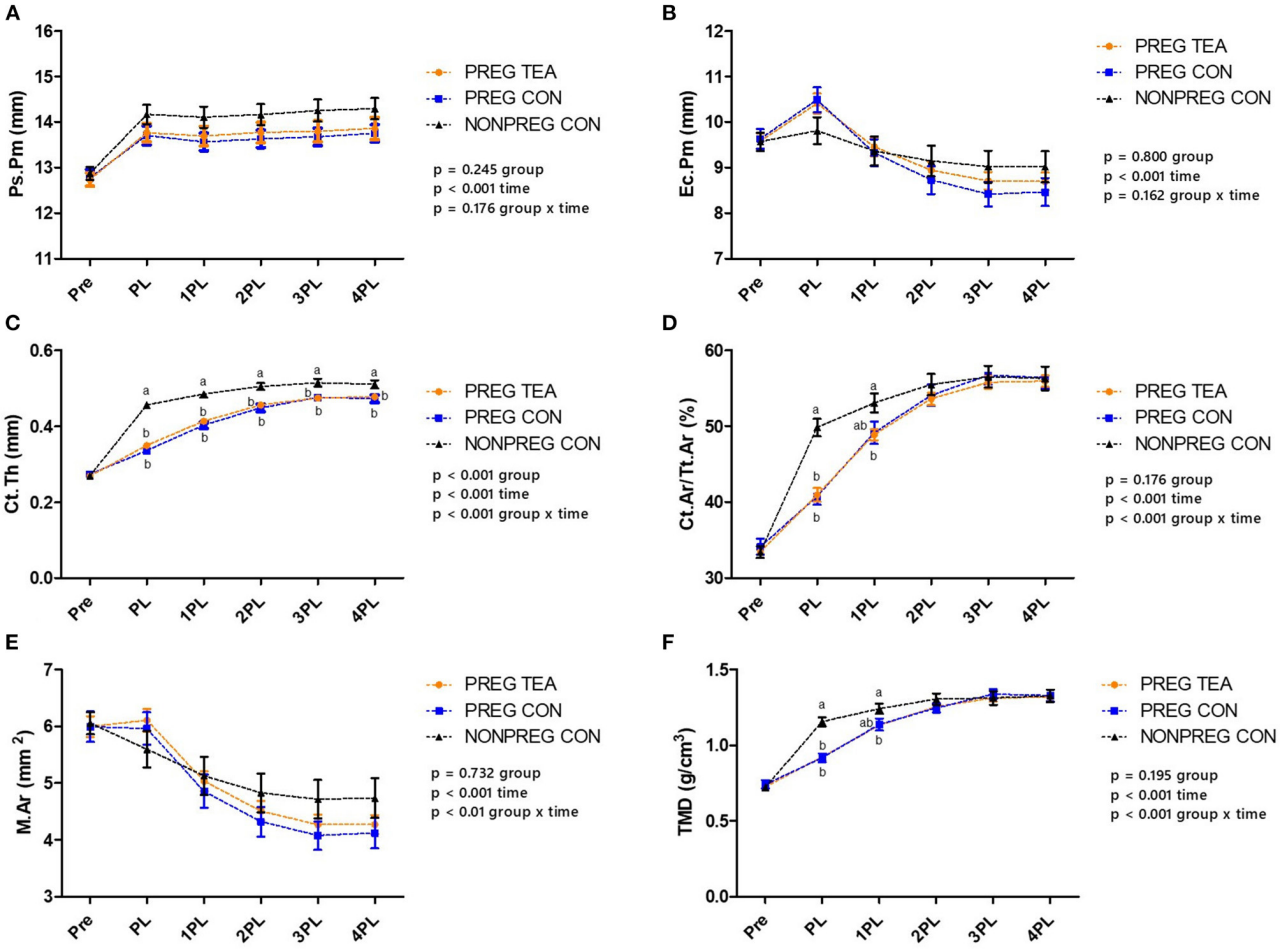
Comparison of cortical bone outcomes between PREG TEA, PREG CON, and NONPREG CON; **(A)** Ps.Pm, **(B)** Ec.Pm, **(C)** Ct.Th, **(D)** Ct.Ar/Tt.Ar, **(E)** M.Ar, and **(F)** TMD. Values are mean ± SEM, *n* = 13 or 14/group. Differing letters denote a significant difference among groups within a time point. Pre: pre-pregnancy, PL, post-lactation; 1PL, 1 month post-lactation; 2PL, 2 months post-lactation; 3PL, 3 months post-lactation; and 4PL, 4 months post-lactation.

Ps.Pm, Ec.Pm, and M.Ar were similar between all groups at all time points (Figures 5, 6A,B,E). However, there were significant (*p* < 0.05) reductions in Ct.Th, Ct.Ar/Tt.Ar, and TMD in PREG TEA and PREG CON following pregnancy and lactation (Figures 6C,D,F). More specifically, Ct.Ar/Tt.Ar and TMD recovered to levels of NONPREG CON by 2PL while Ct.Th remained reduced (*p* < 0.05) in PREG TEA and PREG CON for the remainder of the study.

### Discussion

There were two key findings from this study. The first was that reductions in BMD and structure of trabecular bone following pregnancy and lactation remained at 4 months post-lactation. This finding was unexpected as the majority of rodent studies have reported a complete recovery of BMD following pregnancy and lactation within 4 weeks (1, 33, 34). The few studies that have shown an incomplete recovery in terms of trabecular bone structure measured rats for a shorter recovery period post-lactation (6 weeks) (35, 36) and scanned the tibia weekly - prior to mating through 6 weeks post-lactation–with similar findings to the present study in which BV/TV, Tb.N, and Tb.Sp did not fully recover when compared to non-pregnant control rats (35). The other key finding was that consumption of RR tea consumption supported the recovery of trabecular BMD and structure following pregnancy and lactation though not to the level of non-pregnant control group, but significant improvements in trabecular BMD and structure (BV/TV, Tb.N, and Tb.Sp) were evident by 1 or 2-months post-lactation when comparing PREG TEA to PREG CON. However, our hypothesis that RR tea intervention would result in higher BMD and improved bone structure post-lactation was not proven.

There are several potential reasons why the present findings differ from earlier studies in terms of recovery of trabecular bone. One potential reason is the differing imaging techniques used between the current study and the majority of previous studies. DXA and bone ash weights were formerly the primary methods used which include assessment of the entire bone-both cortical and trabecular portions (37). As well, ash weight is an endpoint measure making it impossible to observe longitudinal changes in bone. The present study used μCT that allowed for the separate analysis of trabecular and cortical bone. As trabecular bone represents approximately 20% of the total skeleton (with cortical bone comprising the remaining 80%) it is possible that significant reductions in trabecular bone will be masked if whole bone BMD is measured. Evidence for this explanation includes similar findings of permanently reduced trabecular bone structure following lactation for other studies in older rats which have also measured bone structure using μCT without reliance on DXA or ash weights to determine BMD or bone mineral content, respectively (35, 36). Another potential reason for the finding of an incomplete recovery of trabecular bone could be the age at which the rats underwent pregnancy and lactation. In the current study, mating began at 8 or 10 weeks (56 or 70 days) of age, which is likely comparable to early adulthood (38). Our findings demonstrated that non-mated rats had increased trabecular BMD and cortical TMD at the PL scan at 15 or 17 weeks of age (compared to their PRE scan at 8 or 10 weeks of age) demonstrating that they were still growing during this period. However, no significant increases in trabecular BMD or cortical TMD occurred after this scan suggesting that maximal mineral content (BMD, TMD) had been reached between the PRE (8 or 10 weeks of age) and PL (15 or 17 weeks of age) scans. Rats may have been challenged in terms of recovery as lactation-induced resorption may have been simultaneously occurring with growth, leading to greater reductions in bone quantity and structure than may have occurred if the rats were older and had reached maximal mineral content. Previously, our lab measured trabecular BMD and cortical TMD by *in vivo* μCT starting at 13 weeks of age through to 25 weeks of age (at 4 week intervals) in female Sprague-Dawley rats and no differences in BMD or TMD were detected between any of the 4 weeks intervals indicating that maximal mineral content (BMD, TMD) had been reached and occurred prior to 13 weeks of age (32). The age at mating (8 or 10 weeks old) in our study was selected as it reflected the age that rats are often bred at commercial and research facilities.

RR tea consumption stimulated a greater recovery of trabecular BMD and bone structure outcomes (BV/TV, Tb.N, and Tb.Sp) by 1 or 2PL, and for most of these outcomes this benefit persisted compared to the group not receiving tea. Though, it is important to consider that outcomes remained lower than the growth control and disproved the original hypothesis. Previously, RR herbal tea has been shown to contain a large quantity of unique polyphenols (aspalathin, aspalalinin, nothofagin) with antioxidant capacity (30, 39). Antioxidants have been shown to decrease ROS supporting osteoblast proliferation, preventing osteocyte apoptosis and inhibiting osteoclast activity (25, 40, 41). This increased quantity of antioxidants provided by polyphenols within RR herbal tea may be able to attenuate the rise in ROS which occurs as a result of pregnancy and lactation leading to greater rates of bone formation, reduced rates of osteoclast resorption, and an overall improved recovery in bone structure and quantity. Although no mechanisms were investigated in this study, previous *in vitro* studies have demonstrated significantly reduced osteopontin gene and protein expression by osteoblasts in response to RR tea which may increase mineralization (16, 17). There were no significant differences in the timing of recovery of cortical outcomes between PREG TEA and PREG CON. This discrepancy is likely due to trabecular bone being more metabolically active making it responsive to acute alterations while cortical bone is less metabolically active and more resilient to acute changes. In a recent study, researchers measured trabecular structure of two distinct sites (tibia and vertebrae) in Sprague-Dawley rats prior to mating through to post-weaning and related trabecular structural outcomes to the proportion of a typical load (36). Trabecular structure (BV/TV, Tb.N, Tb.Sp) in the tibia were greatly reduced but was also found to be responsible for bearing a significantly lower mechanical load than the vertebrae which did not have as large a reduction in BMD and trabecular structure. As well, the researchers observed no difference in compressive properties (peak load, stiffness, and energy to failure) of lumbar vertebrae between rats who did not undergo pregnancy and lactation and rats who were 6 weeks post-lactation, signifying a complete recovery of mechanical strength. The authors propose that these findings may support and explain findings of decreased BMD without any alterations in fracture risk as areas which are more mechanically loaded do not have as severe bone loss and are able to retain their mechanical properties whereas much of the bone loss is localized to areas that are not as mechanically loaded (i.e., tibia).

Despite the need for the tea to be highly concentrated to reach levels which would be comparable to a human drinking approximately 12 cups a day there was no aversion to the taste as *ad libitum* water intake was similar between PREG TEA and PREG CON groups. Organ weight is frequently used as an indicator of overall organ health and safety (42)–and the liver and kidney weights–did not differ between PREG TEA and PREG CON. Moreover, pup weights were similar between PREG TEA and PREG CON during lactation at 3, 7, 14, and 21 days of age. These findings suggest that the intake of RR at the level studied was safe though more detailed analyses would be needed before making a definitive conclusion about safety.

Sprague-Dawley rats are cost effective (compared to clinical models), easily accessible, and provide a good model for initial *in vivo* studies (43). However, there are some key similarities and differences between the current preclinical model implemented and humans which should be stated. Rat litters contain significantly more pups and as a result the demand for calcium is also substantially increased (44). While humans lose 5–10% of their BMD following pregnancy and lactation, rats commonly lose 25–35% (1). Although this does not directly represent the human situation the greater magnitude of change provides an exaggerated model in which potential interventions can be tested. As well, during pregnancy and lactation there are extensive physiological adaptations to provide calcium to offspring and these adaptations are largely similar between humans and rats with the exception of intestinal absorption of calcium during lactation. In humans, skeletal resorption is able to provide enough calcium for their offspring during lactation but due to the greater demand placed on rats by larger litter sizes, intestinal calcium absorption stays elevated during lactation (2, 45).

Strengths of this study include specific methodological aspects which are summarized in Supplementary Material 1. For example, having the same males equally represented in both pregnancy groups, aided in controlling for the influence of paternal genetics. This is important as previous research has demonstrated that the paternal genetics can influence the amount of mineral accrual *in utero* making it an important aspect to be controlled for (46, 47). Furthermore, every dam in each group was mated with a different male reducing the genetic contribution that any one male may have within a group to give a more diverse and realistic group of litters. Additionally, longitudinal *in vivo* measurements within the same rat provided greater statistical power for the study as it controls for many of the variables between subjects. Longitudinal measures can also identify when the ideal timing of an intervention (i.e., consumption of RR tea) may be to elicit the greatest effects. As well, the relatively long duration of the study allowed for statistically significant differences to be observed between the two groups in terms of trabecular recovery. Another strength of this study was the mating strategy used. By staggering the mating, we were able to perform scans at precise times around pregnancy and lactation. For the delivery of tea, we adjusted the concentration on a weekly basis in relation to body weight and intake to allow for *ad libitum* access while still ensuring rats received the appropriate concentration daily to mimic a real-life scenario more closely. Other methodological strengths included culling litters to ensure normalized calcium demand and milk production by dams as well as scanning rats in alternating group order to ensure that if there were any inconsistencies in X-ray transmissions that it would be evenly spread among the groups.

There are also limitations of the present study. Rats were mated at a time in which they were continuing to accumulate mineral and thus may have been particularly challenged in terms of recovery with the lactation-induced resorption occurring simultaneously with growth. Another limitation to this study is that only one concentration of RR tea was used, and that the level administered would require supplementation. Consuming RR tea and/or polyphenols in the form of a supplement would alter its food matrix and may affect their digestion and absorption, changing the compounds which are interacting with bone. However, new techniques are also being developed to increase the bioavailability of polyphenols from supplements including encapsulation of polyphenols within a shell of polysaccharides, cellulose, starch, or proteins to increase bioavailability and possibly their efficacy (48). Although a beneficial effect of consumption was observed at the concentration studied, future studies should assess other concentrations of RR tea to determine if higher concentrations would elicit greater benefits or what the lowest effective concentration would be.

In conclusion, findings from this study demonstrate that consumption of RR tea supported the ability of bone to recover post-lactation in a rat model but did not result in greater BMD and improved structure as hypothesized. Moreover, our unexpected finding that significant reductions in trabecular BMD and structure persisted at 4 months post-lactation provides a basis for more fully understanding the rat model of pregnancy and lactation in terms of bone formation and resorption and should be evaluated further in other rodent models as well as other ages of Sprague-Dawley rats.

## Data Availability Statement

The raw data supporting the conclusions of this article will be made available by the authors, without undue reservation.

## Ethics Statement

The animal study was reviewed and approved by Brock University Animal Care Committee (Protocol #18-03-02).

## Author Contributions

MM and WEW contributed to the conception and design of the study. MM and JY performed the *in vivo* study, sample collection, and analyses, as well as data collection. MM performed the data analysis, statistical analysis, and wrote the first draft of the manuscript. All authors contributed to manuscript revision, read, and approved the submitted version.

## Funding

This research was supported by a Natural Sciences and Engineering Research Council NSERC (05573) Discovery Grant (WEW) and the Canada Research Chairs program (WEW).

## Conflict of Interest

The authors declare that the research was conducted in the absence of any commercial or financial relationships that could be construed as a potential conflict of interest.

## Publisher's Note

All claims expressed in this article are solely those of the authors and do not necessarily represent those of their affiliated organizations, or those of the publisher, the editors and the reviewers. Any product that may be evaluated in this article, or claim that may be made by its manufacturer, is not guaranteed or endorsed by the publisher.
