# Supplementary material for: Pregnancy and Lactation in Sprague-Dawley Rats Result in Permanent Reductions of Tibia Trabecular Bone Mineral Density and Structure but Consumption of Red Rooibos Herbal Tea Supports the Partial Recovery
Source: Front Nutr. 2021 Dec 7;8:798936. doi: 10.3389/fnut.2021.798936 (PMC8689395; doi:10.3389/fnut.2021.798936)
Supplement: Supplementary file 1 [file Data_Sheet_1.docx]

**S1. Methodological considerations within this study**

| Methodological Aspect | Explanation |
| --- | --- |
| Mating strategy (see S3) | There was equal representation of each of the males between both groups to help control for potential paternal effects.  Each male only provided one litter to each group as opposed to using the same male to produce multiple litters. This helped to control for a potential paternal effect.  Because of time involved with *in vivo* scanning and repeated, longitudinal measurements, staggering the mating allowed us to perform scans at precise times around pregnancy and lactation. |
| Culled litters | Litters were culled to equalize maternal milk production and thus normalize the calcium demand on dams. |
| Longitudinal assessment of BMD, TMD and trabecular and cortical bone structure | Reduces internal variability as the same rat is scanned at multiple time points.  The longitudinal assessment reduces the numbers of rats needed within a group. |
| Delivery of tea (see S2, S4) | Adjusted the RR tea concentration to allow *ad libitum* consumption of RR tea polyphenols while maintaining desired daily intake based on body weight and daily water intake.  TPC was measured throughout the study to ensure preparation of RR tea was consistent over the duration of the study. |
| Scanning strategy | At each scan time, rats were selected to be scanned in an alternating group order to ensure similar scanning conditions among groups, including potential variation in X-ray transmission among the multiple scanning days. |

**S2. *A priori* sample size calculation**

*Sample size analysis was conducted using data from a previous study which evaluated the ability of green tea polyphenols to mitigate bone loss related to LPS and chronic inflammation. BV/TV was the outcome measure used for sample size analysis as it allows for the detection of longitudinal changes in bone volume.*

**Sample Size Calculation**

Effect size (Cohen’s d):

$$d= \frac{\mathrm{mean}\frac{\mathrm{BV}}{\mathrm{TV}}of treatment-mean\frac{\mathrm{BV}}{\mathrm{TV}} of control}{standard deviation of control}$$

$$d= \frac{23.82-17.54}{3.83}$$

$$d= 1.64$$

α = 0.05

power = 0.80

σ = 2.80 (as a function of α and power)

d = 1.64

$$N=2 \left[ \frac{\sigma}{d} \right]^{2}$$

$$N=2 \left[ \frac{2.8}{1.64} \right]^2$$

$$N=6$$

**S3. Calculation of the concentration of red rooibos used as the intervention, determined through allometric scaling**To achieve a supplemental dose that would be equivalent to 12 cups/day in humans:

- Average amount of red rooibos/cup = 2.08 grams/cup (Weight of red rooibos tea in a commercially purchased tea bag has been previously measured by our lab to be this value)
- 2.08 grams/cup x 12 cups/day = 24.96 grams/day x 1000 mg/gram = 24 960 mg/day
- Reference weight for a human is 60 kg
- Human Equivalent Dose (HED) = 24 960 mg red rooibos/day x 1/60 kg = 416 mg red rooibos/kg of body weight/day
- Conversion factor to convert a HED to an AED (rat) is 6.2 based on allometric scaling
- Animal Equivalent Dose (AED) = HED x 6.2
- AED = 416 mg of red rooibos/kg of body weight/day x 6.2 = (2579 mg of red rooibos/kg of body weight/day) ~ (2600 mg of red rooibos /kg of body weight/day
- AED for rats is approx. 2600 mg of red rooibos/kg of body weight/day OR 2.6 g red rooibos/kg of body weight/day

**S4. Study mating design**
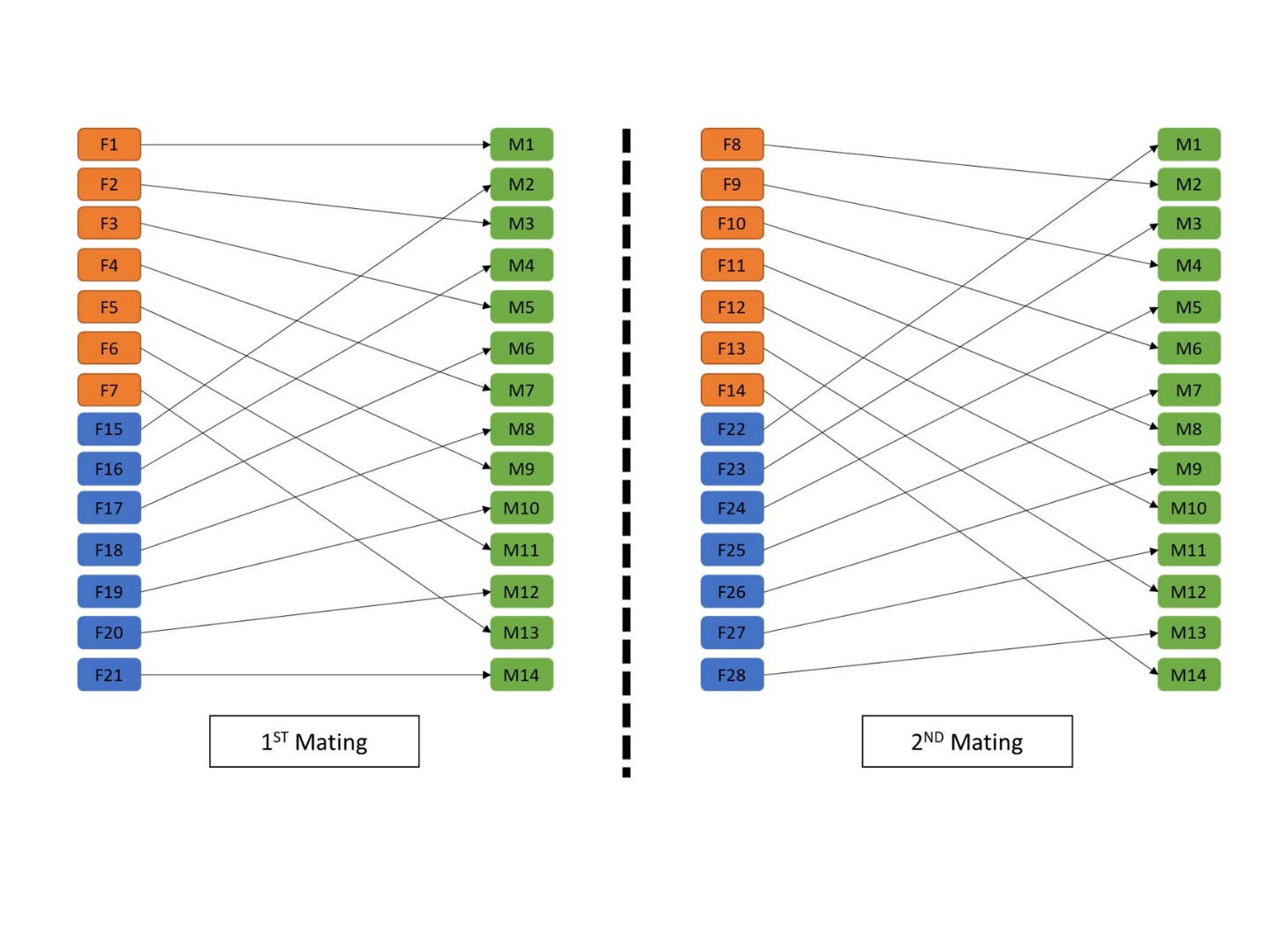

After 3 weeks of acclimatization, half of PREG TEA and PREG CON groups were paired with a male and mated. Two weeks later, and after successful mating (determined by an increase in body weight) the remaining half of each group were paired with a male (2 weeks later) that was previously mated with the opposite group. F: females, M: males, orange squares: PREG TEA, blue squares: PREG CON, green squares: males, and each number represents a unique animal.

**S5. Example calculation for red rooibos concentration**

Average Body Weight of Rat from PREG TEA (Measured during the previous week) = 0.350 kg

Average Daily Water Intake (Measured during the previous week) = 20 mL/day

Desired Daily Concentration of Red Rooibos (Calculated from S2) = 2600 mg of red rooibos/kg of body weight/day

**Calculation**

Desired Daily Concentration of Red Rooibos x body weight = Daily Intake of Red Rooibos

2600 mg of red rooibos/kg/day x 0.350 kg = 910 mg/day

Daily Intake of Red Rooibos ÷ Average Daily Water Intake = Concentration

910 mg/day ÷ 20 mL/day = 45.5 mg/mL

Knowing this information helps to determine how much tea to prepare based on a number of days and number of rats.

**S6. Trabecular task list**The following task list was used to distinguish trabecular from cortical bone in the proximal tibia and analyze the corresponding trabecular BMD and structure outcomes

- Thresholding
  - Adaptive – 2D space
    - Low: 42
    - High: 255
    - Constant: 2
  - Pre-thresholding
  - Kernel: round (radius: 2)
  - Background: dark
- Despeckle
  - Sweep – 3D space
  - Remove: all except largest image
  - Apply to: image
- Morphological operations
  - Closing – 3D space
  - Kernel: round (radius: 2)
  - Apply to: image
- Morphological operations
  - Opening – 3D space
  - Kernel: round (radius: 1)
  - Apply to: image
- Morphological operations
  - Erosion – 2D space
  - Kernel: round (radius: 1)
  - Apply to: image
- Save bitmaps
  - Apply to: image inside ROI
  - File format: BMP (custom subfolder: bitmaps)
  - Copy shadow projection
  - Copy dataset log file
  - Insert scale bar
- Histogram
  - Unit: BMD - 3D space (inside VOI)
- 3D analysis

**S7. Cortical task list**The following task list was used to distinguish cortical from trabecular bone in the proximal tibia and analyze the corresponding cortical TMD and structure outcomes

- Thresholding
  - Global
    - Low: 63
    - High: 255
- ROI shrink-wrap
  - Mode: shrink-wrap – 2D space
  - Stretch over holes (diameter: 8 pixels)
- Morphological operations
  - Opening – 3D space
  - Kernel: round (radius: 4)
  - Apply to: image
- Morphological operations
  - Closing – 3D space
  - Kernel: round (radius: 3)
  - Apply to: image
- Morphological operations
  - Erosion – 2D space
  - Kernel: round (radius: 1)
  - Apply to: image
- Save bitmaps
  - Apply to: image inside ROI
  - File format: BMP (custom subfolder: bitmaps)
  - Copy shadow projection
  - Copy dataset log file
  - Insert scale bar
- Histogram
  - Unit: BMD - 3D space (inside VOI)

2D analysis
